# Conscientious objection as structural violence in the voluntary termination of pregnancy in Chile

**DOI:** 10.3389/fpsyg.2022.1007025

**Published:** 2022-11-03

**Authors:** Adela Montero, Mirliana Ramirez-Pereira, Paz Robledo, Lidia Casas, Lieta Vivaldi, Daniela Gonzalez

**Affiliations:** ^1^Center for Reproductive Medicine and Integral Development of Adolescence, Faculty of Medicine, University of Chile, Santiago, Chile; ^2^Department of Nursing, Faculty of Medicine, University of Chile, Santiago, Chile; ^3^Medium Care Unit, Pediatrics and Pediatric Surgery Service, Hospital La Florida Dra. Eloísa Díaz, La Florida, Chile; ^4^Center for Human Rights, Faculty of Law, University of Diego Portales, Santiago, Chile; ^5^Department of Law Sciences, Law School, Alberto Hurtado University, Santiago, Chile

**Keywords:** conscientious objection, abortion, structural violence, sexual health, reproductive health, sexual and reproductive rights

## Abstract

**Introduction:**

After three decades of the absolute prohibition of abortion, Chile enacted Law 21,030, which decriminalizes voluntary pregnancy termination when the person is at vital risk, when the embryo or fetus suffers from a congenital or genetic lethal pathology, and in pregnancy due to rape. The law incorporates conscientious objection as a broad right at the individual and institutional levels.

**Objectives:**

The aim of the study was to explore the exercise of conscientious objection in public health institutions, describing and analyzing its consequences and proposals to prevent it from operating as structural violence.

**Materials and methods:**

This study uses a qualitative, post-positivist design. At the national level, according to the chain technique, people who were identified as key actors due to their direct participation in implementing the law were included. Grounded theory was used to analyze the information obtained through a semi-structured interview. The methodological rigor criteria of transferability or applicability, dependability, credibility, auditability, and theoretical-methodological adequacy were met.

**Results:**

Data from 17 physicians, 5 midwives, 6 psychologists, 8 social workers, 2 nursing technicians, and 1 lawyer are included. From an inductive process through open coding, conscientious objection as structural violence and strategies to minimize the impact of objection emerge as meta-categories. The first meta-category emerges from the barriers linked to the implementation of the law, the infringement of the rights of the pregnant person, and pseudo conscientious objection, affecting timely and effective access to pregnancy termination. The second meta-category emerges as a response from the participants, proposing strategies to prevent conscientious objection from operating as structural violence.

**Conclusion:**

Conscientious objection acts as structural violence by infringing the exercise of sexual and reproductive rights. The State must fulfill its role as guarantor in implementing public policies, preventing conscientious objection from becoming hegemonic and institutionalized violence.

## Introduction

Conscientious objection in health is considered the main barrier to accessing legal abortion services (Faúndes and Shah, 2015). Recently it has been contemplated as a non-monetary conflict of interest, the ethical problem arising when the professionals’ interest influences or prevents the fulfillment of their duties, the consequence of which is a service that is not governed by the usual standard but by the non-monetary interests of the staff or the institution (Giubilini and Savulescu, 2020).

### Conscientious objection in the Chilean legal framework

After 28 years of absolute criminalization of abortion, on 14 September 2017, Law 21,030 was enacted in Chile, decriminalizing voluntary termination of pregnancy (VTP) on three grounds. Ground No. 1: The person is at vital risk so that the termination of pregnancy avoids danger to her life; ground No. 2: The embryo or fetus suffers from an acquired congenital or genetic pathology of lethal character, incompatible with independent extrauterine life; ground No. 3: The pregnancy is the result of rape, provided that no more than 12 weeks of gestation have elapsed in those older than 14 years, and 14 weeks in those younger than 14 years (Ministry of Health Chile, 2017).

The law marked an inflection point for conscientious objection by incorporating it as a right for the physician required to perform the termination and by extending it to all professional and technical personnel working in the surgical ward during the termination of pregnancy, including health institutions with or without confessional ideology^1^ (Ministry of Health Chile, 2017; Montero and Villarroel, 2018; Tribunal Constitucional de Chile, 2019b).

After the enactment of the law, the debate on conscientious objection continued. Before the end of President Bachelet’s second term in office (2014–2018), a specific protocol was drawn up to regulate its exercise following the guidelines established in the law (Ministry of Health Chile, 2018c). With the advent of a new government with a conservative political ideology that was against abortion, modifications were introduced to this protocol, reducing the restriction for its invocation by individual and institutional providers (Ministry of Health Chile, 2018b). After analyzing the proposal, the Office of the Comptroller General of the Republic^2^ rejected the new protocol, considering that private institutions that provide gynecological-obstetric services and receive State support could not declare themselves to be objectors, mandating the preparation of a specific regulation on this matter. The regulation mentioned before came into force on 23 October 2018 (Ministry of Health Chile, 2022c), following the unconstitutionality appeal filed by parliamentarians opposed to abortion (Tribunal Constitucional de Chile, 2019a) and the new ruling of the Constitutional Court, which allows private health institutions with State contributions to declare themselves objectors even when they perform functions subrogated by the State^3^ (Tribunal Constitucional de Chile, 2018). The regulation stipulates that public health institutions may not declare themselves objectors (Ministry of Health Chile, 2022c).

The broad scope of conscientious objection in Chilean law reveals the complexity of its exercise, raising concern about the risk that it may operate as an ideological barrier affecting the sexual and reproductive health and rights of pregnant persons^4^ in the implementation of this public policy (Montero and Villarroel, 2018; Montero et al., 2021).

In this context, according to information updated to 31/03/2021, obtained from 57 public health institutions, it shows that out of 4,378 contracted employees, 12.7% object on ground 1 (risk to the life of the pregnant person), 17% on ground 2 (lethal congenital alteration), and 25.7% on ground 3 (pregnancy due to rape). According to the profession, 19% of obstetricians/gynecologists objected on ground 1, 27.6% on ground 2, and 49% on ground 3 (Montero et al., 2021).

### The health system in Chile

In order to contextualize conscientious objection and access to VTP, we must detail some characteristics of the health system.

Chile has a hybrid system consisting of public insurance or the National Health Fund (FONASA), which currently covers about 15 million beneficiaries, equivalent to 77% of the population, and consists of four levels (A, B, C, and D), with the most vulnerable people concentrated in levels A and B (National Healthcare Fund, 2022). It includes a private system administered by private health insurance companies (ISAPRES), private entities based on health insurance, created during the Chilean civil-military dictatorship, which involves about 19% of the population (The Isapres, 2022). Other specific insurances for officers of the armed forces are financed with general taxes and other private non-profit or mutual health institutions, with coverage for occupational accidents and diseases (Becerril-Montekio et al., 2011).

According to the degree of complexity, health services are provided at three levels of care. In the public sector, the first level corresponds to primary healthcare (PHC), which is responsible for comprehensive care according to the life course through health promotion, prevention, treatment, rehabilitation, and palliative care (World Health Organization, 2022). It is mainly managed by municipal health directorates and corporations,^5^ with regulation and supervision by the Ministry of Health (Becerril-Montekio et al., 2011; Dazarola, 2022; Goldstein, 2022). The secondary and tertiary levels are administered by the Health Services, under the Ministry of Health, and include specialized outpatient and inpatient care (Becerril-Montekio et al., 2011; Goldstein, 2022). In the private sector, the levels are limited to care provided by a provider in medical centers or clinics.

According to the technical standards of the Ministry of Health, services directly related to VTP, such as the establishment of grounds,^6^ psychosocial accompaniment, and procedures for termination of pregnancy, are contemplated at the obstetric and gynecological specialty level and are therefore only provided at the secondary and tertiary levels of care^7^ (Ministry of Health Chile, 2018a).

### Structural violence in health

Structural violence refers to violence in which some social structure or institution can harm people by preventing them from satisfying their basic needs and focuses attention on the legal, political, economic, and sociocultural systems and relationships that are part of society and that shape the experiences of individuals, including health and wellbeing (Sinha et al., 2017).

Concerning VTP, these structures or institutions would correspond, on the one hand, to the State by incorporating a broad conscientious objection and establishing it as a right. On the other hand, it would include health institutions where this objection is operationalized.

Another example of the violation of rights was the debate surrounding emergency contraception. Chile has a long history of sexual and reproductive health policies. In 1931, a Sanitary Code was enacted, which permitted abortion on therapeutic grounds. In 1967, the government of Eduardo Frei Montalva adopted the first fertility regulation policy and the first sex education program, policies that the government of Salvador Allende continued. The civil-military dictatorship reformed the health system and forbade therapeutic abortion in 1989. With the recovery of democracy, new problems emerged that hindered the advancement of sexual and reproductive rights (Cubillos, 2019). One of them has been the controversy regarding emergency contraception, where the public policy found opponents to its distribution in 2007. A pharmaceutical chain and several majors—in charge on primary healthcare—invoked conscientious objection to selling or providing emergency contraception (Casas, 2008). The enactment in 2010 of Law 20,418 named Sets Standards on Information, Guidance, and Services Regarding Fertility Regulation resolved the contradiction of access to emergency contraception. The law has no reference to conscientious objection (Ministry of Health Chile, 2022b). However, the national norms on fertility regulation considers the possibility that if there are conscientious objections, the provider must refer, without delay, the person to another professional (Ministry of Health Chile, 2006).

These structural inequalities are especially detrimental to women due to the intersection of gender and existing conditions, such as poor health, inadequate education, and access to continuity of care (Nandagiri et al., 2020), giving rise to unequal life opportunities that affect the health and wellbeing of women throughout their life cycle.

Considering that in Chile, the majority of the population receives healthcare in public health institutions (Becerril-Montekio et al., 2011; National Healthcare Fund, 2022), this article aims to explore the exercise of conscientious objection within these institutions, describing and analyzing the main consequences of VTP and the proposals to prevent it from operating as a form of structural violence against pregnant persons. Another aim of this article is to propose orientations and guidelines that contribute to solving and preventing the difficulties observed in implementing this public policy.

## Materials and methods

### Design

The research design corresponds to a qualitative one under the post-positivist paradigm, positioning the researcher as a relative observer, accepting the inability to reach an absolute objective State and a complete understanding with only approximations to reality being feasible to obtain. The researchers may not be able to fully understand what it is or how to get to reality because of hidden aspects (Lincoln and Guba, 2017).

### Recruitment of participants and collection of information

The selection of participants was made considering the public health institutions at the second and third levels of healthcare, mandated according to the technical standard to perform VPT (Ministry of Health Chile, 2018a). In health institutions where it was possible to obtain the director’s authorization, initial contact was made with a key informant. The informant made the contact possible through snowball sampling (Kirchherr and Charles, 2018) to those professionals who were directly involved in the implementation and application of Law 21,030: personnel in managerial positions, members of the psychosocial accompaniment team (psychosocial duo),^8^ and members of the biomedical health team (obstetricians and gynecologists, anesthesiologists, midwives, and nursing technicians). The key informants received personalized requests as invitations informing them about the study. In addition, civil society actors^9^ were incorporated.

The data collection technique was semi-structured interviews. A protocol was prepared, validated by expert judgment and pilot tests for making the adjustments, allowing the incorporation of the main thematic areas related to conscientious objection in the implementation of the VTP law: characteristics and process for its invocation and exercise, role of managers, knowledge of objectors’ identity, implications for the care of the pregnant persons, and proposals and strategies that guarantee the right to dignified, effective, and timely care.

Although the protocol has standard guidelines for different participants, some aspects apply to everyone according to their role within the institution. Different protocols were used according to the type of relevant actors.

A total of two authors conducted interviews with proven experience in this technique. The interviews were conducted in person and by videoconferencing due to the pandemic of SARS-CoV-2. After the interview, only the audio recording was transcribed into text by two transcribers who signed a confidentiality agreement.

### Data analysis

The qualitative data analysis was conducted using grounded theory according to Strauss and Corbin (Bryant and Charmaz, 2019), making it possible to describe and explain the content and internal structure of a partial or insufficiently studied phenomenon such as the one proposed. The analysis includes open coding, constructing codes from particular data, and giving rise to categories and meta-categories (Chun Tie et al., 2019).

Before the analysis, it was verified that the transcription of the interviews into text was literal. The transcriptions were read several times to obtain a general understanding of their content. Codes were compared in terms of similarities and differences. Codes with similar meanings were assigned to a category.

According to the constant comparison method, the initial categories were compared and integrated, achieving a common category. In the abstraction stage, the categories and subcategories were labeled according to the codes and their contents, obtaining the content of the category. In addition, the analysis was supported by ATLAS.ti version 9.0.5^®^ software.

To ensure the quality and validity of the research, the criteria of methodological rigor of transferability or applicability, dependence, credibility, auditability, and theoretical-methodological adequacy were met (Rojas and Osorio, 2017).

To ensure transferability, a sociodemographic survey was applied in order to gather information from the participants, which would allow other researchers to apply this information in their own contexts. Dependability was achieved through triangulation of the analysis by the researchers. Credibility was achieved through an exhaustive process both in the methodological design and in the fieldwork and analysis, incorporating the notes obtained during the data collection process (memos). Auditability was obtained through the rigorous transcription of the interviews and a detailed description of the methodological path. Theoretical-epistemological adequacy was used as the last criterion (Rojas and Osorio, 2017). After a review of various paradigms and perspectives by the research team, it was decided to use grounded theory to be consistent with post-positivism (Lincoln and Guba, 2017).

### Ethical implications

Ethical implications are related to the participants’ protection and risk-to-benefit ratio, particularly about discussing sensitive subjects. Per Chilean regulations on research ethics, prior to recruitment, authorization was requested from the hospital director^10^ (Ministry of Health Chile. Department of Public Health, 2022).

The participants’ authorization was required for the recording in an audio format and subsequent transcription to text. The participants’ right to suspend the interview or withdraw from the study when they considered it pertinent was made explicit, including the non-inclusion of the information provided in the processing or analysis phases, without having to justify their decision.

Informed consent was obtained from the participants prior to the interviews, which were conducted in a safe space in agreement with the participants to avoid interference and to safeguard confidentiality, which was also protected by encrypting all audio files and the transcription into text, with a password available only to the team of researchers and transcribers. The information from the interviews was anonymized so as not to identify the participants and to avoid linking them to the health facility from which they came. The identity of those who participated is only known to those who conducted the interviews.

This research was approved by the Ethics Committee for Research on Human Beings, Faculty of Medicine, University of Chile (Act No. 009 - 2020).

## Results

From January 2021 to January 2022, 39 interviews were conducted with key informants from eight public health institutions in Chile and two from civil society institutions, reaching information saturation.

The participants were 13 members of the psychosocial accompaniment team (psychosocial duo), 11 officials in managerial or coordination positions, 11 members of the biomedical health team, and 4 civil society representatives.

According to profession, the study included 14 obstetricians/gynecologists, 1 anesthesiologist, 1 public health specialist, 1 neonatologist, 5 midwives, 6 psychologists, 8 social workers, 2 nursing technicians, and 1 lawyer.

The average age of the participants was 43 years (24–66 years); the average work experience was 17.5 years (1–41) years; 72% of them were female. Interviews lasted an average of 55 min (23–150 min).

From the analysis of the information, the following codes emerged directly from the narrative of the participants: refusal to participate indirectly in the VTP, lack of justification for conscientious objection, lack of knowledge of objectors’ identity, obstructive expert committees, dissuasive and erroneous information, doubting the story, inducing change in the decision of the pregnant person, delay in the constitution of grounds, false conscientious objection, conscientious comfort, readjust shifts, substitute functions of conscientious objectors, procure healthcare by non-objecting professionals, justification and transparency of the objection, limit the objection to actions directly involved in VTP, professional engagement, respect diversity of thought, local protocolization, committees of expert facilitators, role as guarantor of users’ rights, role as facilitator of implementation, training on the content of the law, addressing myths and apprehensions, addressing conscientious objection, bioethics training, labor choice of objectors, limiting objectors in the public sector, system for monitoring the implementation of the law, and eliminate individual and institutional conscientious objection (Table 1).

**TABLE 1:** Emerging categories and meta-categories.

Meta-category	Category	Codes
Conscientious objection as structural violence	Barriers to the implementation of the law	– Refusal to participate indirectly in the VTP – Lack of justification for conscientious objection – Lack of knowledge of the identity of objectors – Obstructive expert committees
	Infringement of the rights of the pregnant person	– Dissuasive and erroneous information – Doubting the story – Inducing change in the decision – Delay in the constitution of grounds
	Pseudo conscientious objection	– False conscientious objection – Conscientious comfort
Strategies for minimizing the impact of conscientious objection	Reorganize the healthcare	– Readjust shifts – Substitute functions of conscientious objectors – Procure health care by non-objecting professionals
	Regulating the practice	– Justification and transparency of objection – Limit the objection to actions directly involved in the VTP. – Professional engagement – Respect diversity of thought – Local protocolization – Committees of expert facilitators
	Management roles	– Role as guarantor of users’ rights – Role as facilitator of implementation
	Training	– Training on the content of the law – Addressing myths and apprehensions – Addressing conscientious objection – Bioethics training
	Ensuring compliance with the law	– Labor choice of objectors – Limiting objectors in the public sector – System for monitoring the implementation of the law – Eliminate individual and institutional conscientious objection

These codes were grouped into eight categories: barriers to the implementation of the law, infringement of the rights of the pregnant person, pseudo conscientious objection, reorganize the healthcare, regulating the practice, management roles, training, and ensuring compliance with the law (Table 1).

To conclude the open coding, the analysis gave rise to two meta-categories: conscientious objection as structural violence and strategies for minimizing the impact of conscientious objection (Table 1).

The categories and meta-categories that emerged from the analysis of the participants’ discourses are described in the following text.

## Categories

### Barriers to the implementation of the law

The participants identified several obstacles related to the exercise of conscientious objection that acts as a barrier to healthcare for the pregnant person. This category emerges from the following codes: refusal to participate indirectly in the VTP, lack of justification for conscientious objection, lack of knowledge of the identity of objectors and obstructive expert committees.

The refusal of the members of the health team is related to their participation in any indirect action related to the VTP process, such as participating in the constitution of the ground, self-exclusion from the diagnostic process, for example, in the case of fetal non-viability, providing orally administered abortion-inducing drugs, or providing supplies in the ward (gauze and compresses). As one participant points out, diagnosis is inherent to healthcare, so refusal to participate is tantamount to a lack of service.

Another obstruction is lack of justification for conscientious objection,^11^ allowing objection for convenience or false objection, as described here. According to the participants, if it were a requirement to explain and justify the objection clearly, the number of objectors would probably decrease.

The participants, in a significant number of the institutions included in this study, mentioned that the identity of conscientious objector professionals and technicians is not public. In these circumstances, the psychosocial team is forced to infer or assume that the health team members are objectors, essentially through their behavior and attitudes regarding VTP. The importance of recognizing who are objectors lies in providing continuity of care to the pregnant person, avoiding referral to objectors to avoid inappropriate and persuasive treatment and other complex situations that delay care or affect the freedom to decide about the VTP.

In some institutions, the creation of or consultation with *expert committees* appears to hinder the constitution of the grounds. The referral and subsequent discussion of the person’s situation as a clinical case with these committees result in the presentation of arguments and personal positions as objectors, delaying and/or blocking access to timely care. As a member of the psychosocial team points out, one example was the referral of a woman’s case to the ethics committee of an institution.

Some quotations that show evidence of this category exists:

*“We encountered the case of colleagues who wanted to withdraw absolutely from anything that had to do with the care of women who requested a VTP (.) Physicians have no reason to object to the diagnosis we make, it is implicit in our profession to make a diagnosis” (E5: Obstetrician/gynecologist, chief of service).*

*“One of the things that at the beginning was a little difficult with the doctors, it did not matter so much, whatever the ground, as they were reluctant to raise the ground, to put in the record the ground is raised, especially in the third ground” (E24: Supervising midwife).*

*“They have told us: ‘and who is going to give the patient the pills?’ (.), or ‘I cannot go in to give the compresses or gauze to the gynecologist in the ward”’ (E18: Obstetrician/gynecologist, member of the health team).*

*“The part of conscientious objection that is a piece of paper with a signature, without giving any explanation, is not asked (.). If we were stricter with conscientious objection, probably many would not have a real justification for doing so” (E18: Obstetrician/gynecologist, member of the health team).*

*“We do not know who are conscientious objectors, but over the time we have been working we have already identified them and therefore we also try to get closer and do more teamwork with those who are not conscientious objectors because they are the ones who have the disposition, the skills they have been developing, little by little softer skills to be able to work better with the patients” (E9: Psychologist, psychosocial duo).*

*“[The expert committees] (.) these intermediate bodies created ad hoc to obstruct, without being conscientious objectors, are there, they take different forms in the different services, in the different hospitals and I think it is unusual” (E16: Obstetrician/gynecologist, guild association).*

*“(.) the treatment of this person is being in the XXXX hospital, because in my hospital there is no treatment for cancer, radiotherapy I think there is not. So, from XXXX she was referred to the XXXX hospital and there she was seen by the oncologist. This oncologist (.) at least in ground three he is an objector by word, because he is not registered in the paper (.) but he does have an issue with the abortion issue. Then there was another doctor who was the one who fought in the ethics committee so that the woman could have access. In fact, it is a cancer that is very advanced” (E7: Psychologist, psychosocial duo).*

### Infringement of the rights of the pregnant person

This category emerges from the following codes: dissuasive and erroneous information, doubting the story, inducing a change in the decision, and delay in the constitution of grounds.

Although sexual and reproductive rights are a pillar in the struggle for gender equality, allowing women to stop being passive actors and recipients of public policies, empowering them to make decisions in an autonomous and informed manner, as was one of the objectives of the VTP law, in practice, complex situations arise that lead to infringement of the right of individuals to make decisions and access care in an efficient and timely manner. This is evidenced by the fact that objecting physicians provide dissuasive and erroneous information that induces people to change their decision to terminate a pregnancy. This attitude of the objectors also denotes the asymmetry of power manifested by how the way the information is handled.

Regarding the account of the pregnant person in the case of ground of rape, the objectors, regardless of their gender, question and doubt the pregnant person, arguing that the woman may provide unreliable information to achieve an abortion in the face of an unwanted pregnancy.

Instances are described where an objecting physician has intervened directly, pressuring to induce a change in the person’s decision, particularly in grounds 2 and 3, invoking arguments of a religious nature, such as the presence of a miracle that could occur and faith in God. This attitude generates internal conflicts in the pregnant person who questions their decision and the information received from the psychosocial team, for example, regarding fetal non-viability.

Another infringement of the rights of the pregnant person is directly related to the constitution of the grounds for VTP, an act that is delayed by the lack of non-objecting personnel, by requesting extra confirmatory examinations beyond the regulations or by additionally consulting more expert opinions than what is established in the legal norm.^13^

Some quotations that exemplify this category are as follows:

*“The case that was really difficult for me to deal with, a 16-year-old minor, which was very complex (.) It was very bad in this case, the fact that the information was given by a person who was a conscientious objector made certain emphasis that made the patient change her mind (.) This emphasis on the possibility of the risks, of the sequelae that could result, in the end has played against patients who were already well decided and in the end made them change their mind” (E9: Psychologist, psychosocial duo).*

*“There are conscientious objectors, especially in ground three (.), but it was a colleague who said she was going to be infertile, I don’t know if it was a colleague or a midwife, but he told her she was going to be infertile” (E15: Obstetrician/gynecologist, member of the health team).*

*“We have found many barriers, for example, the questioning of the woman, because she went to the rapist’s house, what she was doing there, or that the testimony does not support rape” (E1: Male midwife, NGO accompaniment).*

*“(.) the suspicion of what, ah? that she was wearing short skirts, that she was raped or what? Those comments still occur and the question ‘where were you, and what were you doing?”’ (E35: Obstetrician and gynecologist, VTP coordinator)*

*“One female doctor in particular, who after we had constituted a third ground, took a patient out of the hospital room and took her to tell her what had happened so she could believe her, and that was something that caused us quite a problem, an ethical conflict, and well, that was the only abnormal and worrying situation we experienced with the ground, but we had to make several changes there as well, telling them that it was not their role, to believe or not to believe, but that as public officials they should attend to the patients and if we are telling them and if this woman is saying that she was a victim of rape and this is a pregnancy resulting from rape, it is the right thing to do, she has the right to terminate” (E37: Social worker, psychosocial duo).*

*“It was in ground two, the patient had decided to terminate the pregnancy and she [doctor] approached her to talk to her about her decision (.) and told her to think about it carefully and that she had to have faith, that miracles happen, things like that, so that she would change her decision, so she obviously got very confused and had the feeling that everyone else had lied to her and that there was hope. When it was not real!” (E38: Psychologist, psychosocial duo).*

*“In the institution where I am currently working, there is only one conscientious objector, so the implementation of the law is much more efficient, there will not be a patient who has to go through, I don’t know, several shifts or that there is no one who can perform the abortion, unlike other places where there are days when there is no one who can perform it” (E18: Obstetrician/gynecologist, member of the health team).*

*“Some people in the health professions hinder the establishment of the ground link by asking for extra lab tests*, *for example, if they need a diagnosis, they ask for two diagnoses, if they need a diagnosis from not a specialist, only from an internist or general practitioner, they usually ask for a specialist’s diagnosis, they ask for more tests than necessary, they delay the constitution of the ground” (E39: Psychologist, accompaniment NGO).*

### Pseudo conscientious objection

In this category, the associated codes are false conscientious objection and conscientious comfort. False conscientious objection emerges from the discourses of the participants who in clinical practice identify a covert conscientious objection, for their convenience, out of idleness, without moral support, reactive and arbitrary, evading their professional responsibility.

As a consequence, what the participants call “conscientious comfort” arises, wielded by health officials as a response to the complex values involved in abortion because they do not have the knowledge to diagnose ground 2, to avoid getting involved in legal issues related to ground 3, or as a refusal to carry out administrative activities, such as filling out forms. In short, there is a delay in the woman’s care and an overload of work for non-objectors, as detailed in the following quotations:

*“Those who were making a conscientious objection out of convenience, because we found colleagues who wanted to withdraw absolutely from anything that had to do with the care of women requesting VTP” (E5: Obstetrician/gynecologist, chief of service).*

*“Many colleagues probably did it to avoid getting into trouble and, forgive me if I am wrong, even out of laziness” (E13: Obstetrician/gynecologist, chief of service).*

*“So, he would say ‘no, you know what, I’d rather not have problems and not get involved in this,’ and he would pass the responsibility to someone else and declare himself an objector” (E26: Hospital director).*

*“It is not a glamorous subject, it is not like fetal laser, it is not like echocardiography, it is something that is hard, it is uncomfortable and I think that for many, one of the things that obstructs, perhaps more than conscientious objection is what I call conscientious comfort, in the sense that the doctor sees that it is something complex, a little out of comfort, a little because he does not know, he does not have enough knowledge, then a kind of rotation of patients begins, a kind of pilgrimage of patients who go from one place to another (.) and this is not even conscientious objection, it is convenience, convenience because they do not know about it, it is an uncomfortable issue, we have to fill out paperwork, so we refer them to the other and this also wastes time” (E14: Obstetrician/gynecologist, chief and member of the health team).*

*“The reason of those who signed for the third ground was: why am I going to get into legal problems” (E16: Obstetrician/gynecologist, guild association).*

*“Doctors who are objectors and what I feel is that suddenly they don’t want to take charge of this, it’s like no, so much paper, so many things, so much controversy they generate” (E7: Psychologist, psychosocial duo).*

*“Because the fact of not getting involved in anything at all is very comfortable, and that can’t be, because it also passes the workload unto others” (E5: Obstetrician/gynecologist, chief of service).*

### Reorganize the healthcare

Health teams have had to readjust shifts and replace the functions of objectors with non-objecting personnel, appealing to the companionship and commitment of other professionals. Situations are described in which non-objecting personnel must attend outside working hours to avoid referring the woman to another healthcare center. The psychosocial accompaniment team has also had to seek care from non-objecting staff, with the inconvenience of rescheduling the woman’s care for another opportunity. As it can be seen from the following quotations, the most significant impact of conscientious objection occurs in the case of pregnancy due to rape (ground 3).

*“As in all other places, ground three has been the more complex one here (.) It is a condition in which conscientious objection from the obstetric group has appeared and certain arrangements had to be made for the distribution of shifts so that there would not be any shift with only conscientious objectors for ground three or for any of the other grounds” (E17: Neonatologist).*

*“I have found myself in a situation in which I have had to put together a different team, like ah, I have to do an MVA, so who is the anesthesiologist? oh, you know, it turns out that the anesthesiologist is a conscientious objector. Calling a friend, ‘can you come and help me’ (.) They tell you ‘the nurse who is in that ward is an objector,’ so they will have to find another nurse from another ward (.), or look for a surgical instrumentalist because there are a couple of them who are objectors and do not want to participate. So, things have to be arranged, but it will also depend on whether there is someone who says ‘well, we have to do it’ and not ‘oh no, most of them are objectors, so we’d better refer them”’ (E18: Obstetrician/Gynecologist, member of the health team).*

*“I remember that during the pandemic, I once had to come and place the patient, we did not know that a replacement physician was a conscientious objector and the other physician who was there on the weekend was also an objector and I came on a Saturday to administer the drugs in this situation” (E13: Obstetrician/gynecologist, chief of service).*

*“We have tried to coordinate with those specific doctors, for example, that the patient should not be scheduled for that day, because that doctor is going to be there that day, so he may say no, it is better for this day, because he does not have any problems. So, we try to coordinate everything, to prevent the patient from having to delay so much, that she comes, and then she is hospitalized but nothing is done, so we try to do this so that she also has good care here at the hospital” (E10: Social worker, psychosocial duo).*

### Regulating the practice

According to the participants, it is necessary to substantiate conscientious objection and make the identity of those who invoke it known, limiting it to those who perform a direct and specific action, for example, only to the obstetrician/gynecologist required for the procedure. Similarly, there is a need to promote professional engagement to comprehensive care and respect for women’s autonomy, acting as guarantors of the exercise of this right in the event of possible infringements.

In order to promote the proper functioning of health teams, objection should not act as an obstructing agent, respecting the diversity of thought of its members.

Due to the existence of local particularities in the health institutions that would not be explicit in the general norm (Ministry of Health Chile, 2018a), local protocols and the creation of expert committees as facilitating bodies have been considered to support the constitution of the grounds, without interfering with the right of the pregnant persons to exercise their will freely.

The following quotations reflect this category:

*“I would leave only the real conscientious objectors, in their right to object, very well explained and very well grounded” (E16: Obstetrician/gynecologist, guild association).*

*“It is absolutely transparent, we have no concealment, this information is handled by the midwife manager of the inpatient unit and she has the list of what is happening in terms of conscientious objection, and sometimes there have even been changes by the doctors themselves” (E23: Obstetrician/gynecologist, chief of service).*

*“The other participants in the ward, for example, it seems to me that a surgical instrumentalist or a ward attendant, or a nurse, or a nurse technician that is supporting the anesthesiologist or the anesthesiologist, it seems to me that they should not be conscientious objectors, because the one who performs the medical act in this particular case is the obstetrician/gynecologist” (E13: Obstetrician/gynecologist, chief of service).*

*“There was a doctor who flew the flag and argued in the ethics committee in favor of the possibility of constituting the ground, that it should be what the woman decided” (E7: Psychologist, psychosocial duo).*

*“We have had a team that has worked well, that has worked well on this and I have not seen major difficulties and in fact my colleagues have also been very respectful with those of us who are objectors and who do not participate” (E4: Obstetrician/gynecologist, member of the health team).*

*“We applied the national regulations directly, we did not make any local adaptations. But now we have encountered certain particularities that are making it necessary for us to create a local protocol, basically to have a road map of what to do in each case” (E5: Obstetrician/gynecologist, chief of service).*

*“We set up a small committee to analyze each patient for ground two (.), those who were objectors realized that they could be objectors without the need to become obstructers (.). I think we have achieved at least, I speak for the committee of this institution, a certain maturity. I do not remember that we have had an experience with any woman who has made us see that she feels that this committee has played a role in restricting her exercise of will” (E17: Neonatologist).*

### Management roles

From the interviews, it is clear that managers must ensure compliance with the law, acting as guarantors of patients’ rights and playing a facilitating role in implementing a public policy. One participant’s account reflects situations in which a manager is a conscientious objector and obstructs the rights of clients by refusing to provide care and excluding team members in the discussion of cases.

The participants point out as a fundamental aspect that managers, even if they declare themselves a conscientious objectors, are not exempt from their administrative obligation and must ensure compliance with the law in the institution or unit under their charge. The need to hire non-objecting personnel is also considered within this role.

*“There was a change in the head of the high obstetric risk unit and the doctor who took over as head is very—I think he is the worst of the objectors here—very abusive, not only with regard to VTP issues but also with regard to patients who are admitted for sexual violence and he tries to get them out of the way so as not to attend to them (.) In trying to ensure rights, I think he is one of the obstacles to rights, so when he took over as director, the meetings kind of dissipated, they stopped for a while, and when they were held again, we were never invited again” (E6: Social worker, psychosocial duo).*

*“As a director I am an objector, but I have a tremendously respectful position of the patients’ decision and that I am working in a public system and that this is a patient’s right to decide and my obligation is to comply with the law and make sure it is well complied with” (E14: Obstetrician/gynecologist, chief and member of the health team).*

*“As a hospital director, in that role, I should have gone out to look for obstetricians/gynecologists who were not conscientious objectors, in order to have this service, which is a right for women in our hospital (.). I have a responsibility as director of an institution that in the face of the implementation of a law, which is a right that has been achieved, I have to provide the facilities so that this law can be implemented. I do not have to be a barrier as director, so I have to make my efforts to be able to complement the colleagues I have today so that this law can be implemented in a good way” (E26: Hospital director).*

### Training

The training of health team members is considered another fundamental aspect. The participants pointed out that activities should be included that, in addition to addressing the content of the law, encourage internal discussion, especially regarding the existence of false assumptions (myths) and apprehensions about abortion and the VTP law. One of these false assumptions was the belief that women would saturate health services to have an abortion, which led to the appearance of prejudices on the part of health personnel and a negative predisposition, favoring the invocation of the condition of objectors.

Adequate training could help reduce the number of objectors since there are cases in which objection is invoked because of a lack of understanding of the regulatory framework and the process of implementing the law. The lack of training in ethical aspects is recognized, and training that includes the gender perspective and the rights approach is advocated, promoting humanized and empathetic care by the health team and respecting the autonomy of patients to decide about their own bodies.

*“At the beginning of this law there were too many myths (.), besides the xxxx region is an area that is quite conservative in some sectors, there were many apprehensions. It was one of the places in which there were colleagues who thought that there would be an avalanche of women for ground 3, so we began to demystify it, we began to clarify what conscientious objection meant in itself” (E5: Obstetrician/gynecologist, chief of service).*

*“Many decided to conscientiously object because they did not understand the process very well, and once the process is explained, conscientious objection is no longer” (E1: Male midwife, NGO accompaniment).*

*“We lack a little bit of that, the perinatal obstetric part. We need more education in bioethics” (E13: Obstetrician/gynecologist, chief of service).*

*“Health personnel must be educated in humanizing health, that they should not forget that behind the clinical record there is a real person, that the body is hers, that this is happening to her, that she is the one who has to live with this (.) So I think we need to humanize and start from there and give more emphasis to the rights that women have especially as patients, that the decision in the end is theirs” (E38: Psychologist, psychosocial duo).*

### Ensuring compliance with the law

Some consider a conscientious objection to VTP as a privilege, and therefore, it should be incompatible to the tasks required in the profession, especially in public health institutions, which are responsible for the care of the most vulnerable population, who do not have the possibility of choosing an individual or institutional provider.

In order to ensure adequate compliance with the law, several proposals emerge from the participants, such as the choice of the place of employment of those who object, limiting the number of objectors, establishing a system for monitoring compliance with the law and the elimination of individual and institutional objection in the implementation of public health policies, and not imposing a particular morality that conflicts with access to services related, in this case, to VTP.

*“If we say that conscientious objection is a privilege, when I want this privilege to work in my favor, then I have to leave an institution that provides this service (.)” (E16: Obstetrician/gynecologist, guild association)*

*“In the public system there should not be conscientious objectors, because we can choose where to work, so if you are so committed to your beliefs, go to an institution where everyone agrees with that, that line of thought, because our patients cannot choose” (E18: Obstetrician/gynecologist, health team member).*

*“I would implement a strict surveillance system and a sanction for those public or private institutions that are obstructing the law in any way, because without a sanction it will not work” (E16: Obstetrician/gynecologist, guild association).*

*“If it were within the framework of the law we currently have, I would eliminate institutional and personal conscientious objection, at least in public health, because I believe that public policy cannot depend on the morality of the people who must guarantee access” (E6: Social worker, psychosocial duo).*

## Meta-categories

This section describes the two meta-categories that emerged from the codes presented previously: conscientious objection as structural violence and strategies to minimize the impact of conscientious objection.

The first meta-category, “conscientious objection as structural violence,” derives from the following categories: barriers to the implementation of the law, infringement of the rights of the pregnant person, and pseudo conscientious objection (Table 1). In the process of constant comparison, conscientious objection can be distinguished as a central theme in which the categories mentioned before converge.

In this meta-category, conscientious objection acts as structural violence, supported by the current legislation that allows broad objection for members of the team who participate indirectly in the process of termination of pregnancy.

Structural violence occurs because the institutional system and society allow women’s rights to be infringed by barriers related to access to services guaranteed by the VTP law, such as timely care. In this sense, considering that in the law, there are specific deadlines related to gestational age, as in the ground of rape, if the deadline is exceeded, the person will not be able to terminate the pregnancy, and it is vital that the care be timely and efficient.

In the interviews, the need to have had to take specific measures to avoid a delay in care is described. This delay is manifested by the lack of availability of non-objecting staff and the demand for additional examinations or unnecessary referrals to other specialists to constitute the ground for the interruption.

It also emerges that objectors try to condition the woman’s intention to terminate her pregnancy, by directly obstructing her decision and pressuring her to modify it, imposing their own beliefs in a clinical intervention where power relations are manifested, or even alluding to the occurrence of a divine intervention, as can be seen from the participants’ accounts.

Some health personnel subscribe to conscientious objection without necessarily having a religious or value-based vision that coincides with a moral foundation, giving rise to a false objection or pseudo conscientious objection, which translates into non-compliance with professional duties.

The second meta-category, “strategies for minimizing the impact of conscientious objection,” emerges from the following categories: reorganize the healthcare, regulating the practice, management roles, training, and ensuring compliance with the law (Table 1). It emerges as a response to prevent conscientious objection from acting as structural violence.

It is important to note that this meta-category is derived from the voice of the participants, who are directly involved in the care of pregnant people. The strategies mentioned can be structured into those related to human resources, regulation of healthcare, and administrative management. It is also noted that many of the actions are subject to the willingness of non-objecting personnel and their commitment to women’s rights. Likewise, there is a need for the managers of health institutions to play a more active role as guarantors of rights, promoting a local organization that ensures compliance with the law and focusing on the care of the pregnant person.

The participants question individual and institutional conscientious objection since it constitutes a barrier that underlines the structural violence against women, so some explicitly recommend its annulment within the current legislation.

## Discussion

Abortion continues to be a complex issue, where health, ethical, religious, legal, social, cultural, and political dimensions are intertwined. In Chile, the regulation of abortion (from its absolute prohibition to its recent decriminalization on three grounds) obeys to public interest and political control of life through power relations, which are not always visible. In this sense, in Western societies, the consideration of sexuality only for reproductive purposes and the disqualification of sexual pleasure have been present since ancient times, introducing mechanisms that begin to operate as a device for controlling the population through their sexuality (Foucault, 2013a,b, 2016).

The control of sexuality gives rise to power relations that act strategically in the social environment, influencing the formulation of laws, the State apparatus, and social hegemony, tending to monitor and control the population’s behavior within capitalist society (Foucault, 2013a,b, 2016).

The social control of sexuality, this government of consciences through ideology, across a conservative morality that seeks to control, manage and regulate moral conduct, has contributed to the stigma and criminalization of abortion. Unfortunately, Chile is not the exception, reflected in the positioning of conscientious objection as a right and its extension to health institutions and those who indirectly participate in the procedure of termination of pregnancy, which was raised as one of the main strategies to prevent the enactment of the law.

Regarding conscientious objection, some defend it by considering its derivation from freedom of conscience, understood as the “attribution of having and manifesting the inner convictions on which personal acts are based (.), in accordance with the judgment of one’s own reason by which the moral quality of such actions is recognized, without being disturbed by others or by public authority” (Royal Spanish Academy, 2022). This argument justifies the refusal to participate in ethically reprehensible activities during professional practice, such as the participation and use of scientific knowledge by health personnel in procedures such as torture and other cruel, inhuman, and degrading acts^18^ (Faundes et al., 2013). However, conscientious objection cannot be invoked as a refusal to directly or indirectly perform the actions contemplated within the benefits that have been legally admitted in a country and even less to obstruct the exercise of the right of the population to access them. We consider that here lies the main “problem of conscience” of health personnel, when they impose their personal beliefs or values over their professional duty to promote wellbeing, safeguard the health, and prevent the occurrence of harm to people (FIGO Committee for the Study of Ethical Aspects of Human Reproduction and Women’s Health, 2012; Faundes et al., 2013), particularly those who work in the public service that receives the most vulnerable population.

As Zaami et al. (2021) argue, even though conscientious objection to abortion has been legally recognized by 24 member States of the European Union, the United Kingdom, Norway, and some cantons of Switzerland, the European Court of Human Rights recently established that abortion is a medical act and the State must guarantee access to all persons who legally request it. Sweden is one of the few countries that does not allow conscientious objection. The Swedish court dismisses the admissibility of these situations stating conscientious objection exceeds the respect for the freedom of conscience of health professionals because the individual prerogative cannot be over and above the right to healthcare of those who wish to terminate a pregnancy (Zaami et al., 2021).

As Stuart Mill points out, living in society imposes a line of conduct based on not harming the interests of others, interests that have been recognized as rights by a legal provision or by a tacit agreement (Mill, 2011a). The principle of complete freedom of action is limited to the extent that it only interests and affects one’s own person without harming others. In both cases, a person may harm another by the person’s actions and omissions, responsible for the damage that occurs (Mill, 2011b). This responsibility of those who participate in and oversee healthcare seems to be diluted in the face of conscientious objection, which, as can be seen from our results, contributes to a great extent to infringement of the autonomy and rights of pregnant persons concerning their sexual and reproductive health.

On the other hand, the lower number of cases of VTP than projected and the difficulty in obtaining official information on the prevalence and characteristics of objectors (Montero et al., 2021) prevent us from knowing the real magnitude and consequences of conscientious objection. In Chile during the period 2018–2021, a total of 3,009 cases are registered, with 931 cases for ground 1, 1,536 cases in ground 2, and 542 cases for ground 3 (Ministry of Health Chile, 2022a), figures much lower than the 2,550 cases estimated annually during the legislative processing of Law 21,030 (Castillo and Robledo, 2016).

Regarding the arguments invoked for conscientious objection, previous research reveals that the main motivation derives from a negative evaluation of abortion and religious considerations based on respect for human life from conception to natural death. There are specific arguments for objecting, as of ground 2, where the physician’s commitment to protecting the lives of two patients and considering the fetus as a terminally ill person who deserves palliative care, instead of termination of pregnancy. In ground 3, the majority of conscientious objections invoke arguments without moral support, derived from mistrust of the pregnant person who is a victim of sexual violence and the consideration of the embryo/fetus as a human being without biomedical pathology that justifies the abortion, perceiving it as an innocent victim of this violence (Montero and Ramírez–Pereira, 2020; Ramírez–Pereira and Montero, 2021).

As the results presented here show, even though there are public health institutions that have tried to regulate their exercise, there are others where power relations are perpetuated, where conscientious objection operates as a denial of service, obstructing the implementation of the law and infringement of the right of a pregnant person to quality, timely, and efficient care.

Consequently, based on the findings of our research and the incorporation by the State of conscientious objection as a right and its extension to other actors and health institutions (Ministry of Health Chile, 2017), we can conclude that the current exercise of conscientious objection in Chile, by interfering with the duty of care of health personnel and the right of individuals to VTP, would operate as a form of structural violence, acting as invisible violence, which responds to systemic processes with negative effects on the wellbeing and freedom of pregnant people, causing dissatisfaction of their needs (La Parra and Tortosa, 2003; Farmer et al., 2006). In short, the exercise of conscientious objection, by acting as a barrier, would act as a hegemonic, hidden, indirect, and institutionalized violence derived from power relations that affect decision-making and infringement of the right to access the legally established benefits related to VTP.

It is essential to prevent conscientious objection from becoming a control device that acts as a barrier, contravening the autonomy of the pregnant person, limiting their right to safe termination of pregnancy, infringement of their dignity as a person, and exposing them to harm to their health and life. Therefore, strategies should consider objection as a limited and exceptional resource. One of them would be to appeal to the moral conscience of those who declare themselves objectors, inviting them to reflexively consider, as an examination of conscience, their official duties and their work assignment, especially in the public service, responsible for providing care to most of the population of the country. It is also relevant to contribute to the social destigmatization of abortion, by training future professionals and current members of health teams, including the acquisition of competencies not only regarding the content of the law but also in the incorporation of skills that allow a more humanized and empathetic approach, which allows them to understand the issue of abortion beyond a binary perspective—referring to the imposition or prohibition—and respecting the self-determination of the pregnant person to decide freely on transcendental aspects of their biography, their health, and their life, according to their own convictions, beliefs, and values.

Finally, together with reviewing the imposition of conscientious objection in health as a right, we consider it urgent to regulate and supervise, free of ideological influences, its exercise in the health public and private sector. Bearing in mind the historical period Chile is going through, tending to reformulate its Political Constitution, the State, through its institutions, must fulfill its role as guarantor of the fundamental rights of those who live in a certain territory, especially of the most vulnerable people. Otherwise, it will incur as an actor responsible for the perpetuation of structural violence. Even though organized civil society has a preponderant role in defense of sexual and reproductive rights, we ratify the approach to the obligations of the State toward citizenship (Montero et al., 2017; Montero and Villarroel, 2018).

## Data availability statement

The datasets presented in this article are not readily available because, as approved by the Ethics Committee and the commitment to the participants to protect their confidentiality, the interviews cannot be shared. Requests to access the datasets should be directed AM, amontero@uchile.cl.

## Ethics statement

The studies involving human participants were reviewed and approved by the Ethics Committee for Research on Human Beings, Faculty of Medicine, University of Chile. The participants provided their written informed consent to participate in this study.

## Author contributions

AM and MR-P made substantial contributions to the conception and design of the work, conducted the interviews, drafted the manuscript, and agreed to be accountable for all aspects of the work in ensuring that questions related to the accuracy or integrity of any part of the work are appropriately investigated and resolved. All authors participated in the coding process, analysis, interpretation of the data, critically reviewed the manuscript, provide approval for publication of the content, and contributed to the article and approved the submitted version.

## Footnotes

1 The Constitutional Court, in an instance prior to the enactment of the law, extended conscientious objection to health institutions and to professional and technical personnel working in surgical wards.

2 Office of the Comptroller General of the Republic: It is the highest control body of the State Administration, which is provided for in the political Constitution and is autonomous from the Executive Branch and other public bodies. It controls the legality of administrative acts and safeguards the correct use of public funds.

3 The subrogation of functions by the State implies the delegation of functions to private health institutions, which, through a State stipend, provide health care services.

4 For the purposes of this article, although law 21,030 does not make it explicit, pregnant persons are understood as those who identify with the female gender and those who, even when they do not identify with this gender, have reproductive organs that allow them to gestate, for example transgender men and persons with non-binary gender identity.

5 Chile is territorially and administratively divided into communes, provinces and regions. The municipalities are bodies responsible for communal government and development. Although they have autonomy in their attributions and legal functions, they are part of the State administration (Dazarola, 2022).

6 The constitution of the ground includes all the procedures that accredit that the pregnant person is in one of the grounds contemplated in the law.

7 With regard to VTP, the secondary level in the public sector corresponds to outpatient care in High Obstetric Risk Polyclinics (HOR) integrated or attached to hospital facilities. For private providers, it corresponds to consultations attached to clinics and/or medical centers. The tertiary level corresponds to inpatient care in public sector hospitals and in medium and high complexity private clinics in the private sector (Ministry of Health Chile, 2018a).

8 Law 21.030 establishes the person’s right to access a voluntary accompaniment program, which includes shelter and psychosocial support during and after the decision-making process, which must be carried out with her authorization, in a personalized manner and respectful of her decision. In public health institutions, the accompaniment is carried out by a “psychosocial duo” formed by a professional psychologist and a social worker. The law considers that non-profit civil society organizations may also provide this accompaniment, it being the person’s prerogative to choose the one she considers most appropriate according to her situation and personal convictions (Ministry of Health Chile, 2017, 2018a).

9 Civil society actors are professionals affiliated with a non-governmental organization (NGO) who have participated in the psychosocial accompaniment or training on the law or are affiliated with a professional or guild association.

10 Law 20,120 establishes that: “All biomedical scientific research must have the express authorization of the director of the establishment in which it is carried out”.

11 According to the current regulations, conscientious objection can be invoked without the need for argumentation. The only requirement is to fill out and sign a form with some socio-demographic information and indicate the grounds for objection, being able to declare oneself an objector on all grounds.

13 For the constitution of the grounds, the law establishes the following: In ground 1, the respective medical diagnosis must be available; it does not indicate that other specialists must be consulted. In the case of ground 2, there must be two specialist medical diagnoses (understood as obstetricians/gynecologists). In ground 3, a health team will confirm the concurrence of the facts and the gestational age.

18 Only on 11 November 2016, Law 20,968 which criminalizes the crimes of torture and cruel, inhuman and degrading treatment, was enacted in Chile.
